# Secreted Metabolites from *Pseudomonas*, *Staphylococcus*, and *Borrelia* Biofilm: Modulation of Immunogenicity by a Nutraceutical Enzyme and Botanical Blend

**DOI:** 10.3390/microorganisms12050991

**Published:** 2024-05-15

**Authors:** Dina Cruickshank, Debby E. Hamilton, Ifeanyi Iloba, Gitte S. Jensen

**Affiliations:** 1 NIS Labs, 807 St. George St., Port Dover, ON N0A 1N0, Canada; dina@nislabs.com; 2 Researched Nutritionals, P.O. Box 224, Los Olivos, CA 93441, USA; dhamilton@researchednutritionals.com; 3 NIS Labs, 1437 Esplanade, Klamath Falls, OR 97601, USA; ifeanyi@nislabs.com

**Keywords:** cytokines, interleukin-1β, interleukin-6, interleukin-10, immune activation, inflammation, macrophage inflammatory protein-1-alpha, persister cells, tumor necrosis factor-alpha

## Abstract

Bacterial biofilms are hardy, adaptable colonies, evading immune recognition while triggering and sustaining inflammation. The goals for this study were to present a method for testing the immunogenicity of secreted metabolites from pathogenic biofilm and to document whether biofilm treated with a nutraceutical enzyme and botanical blend (NEBB) showed evidence of reprogrammed bacterial metabolism, potentially becoming more recognizable to the immune system. We screened immune-modulating properties of metabolites from established biofilm from *Pseudomonas aeruginosa* (Pa), *Stapholycoccus simulans* (Ss), and *Borrelia burgdorferi* (Bb). Secreted metabolites significantly increased the cytokine production by human peripheral blood mononuclear cells, including Interleukin-1-beta (IL-1β), Interleukin-6 (IL-6), macrophage inflammatory protein-1-alpha (MIP-1α), tumor necrosis factor-alpha (TNF-α), interleukin-1 receptor antagonist (IL-1ra), and interleukin-10 (IL-10). Pa metabolites triggered the most robust increase in IL-1β, whereas Bb metabolites triggered the most robust increase in IL-10. NEBB-disrupted biofilm produced metabolites triggering altered immune modulation compared to metabolites from untreated biofilm. Metabolites from NEBB-disrupted biofilm triggered increased MIP-1α levels and reduced IL-10 levels, suggesting a reduced ability to suppress the recruitment of phagocytes compared to untreated biofilm. The results suggest that nutraceutical biofilm disruption offers strategies for inflammation management in chronic infectious illnesses. Further clinical studies are warranted to evaluate clinical correlations in infected human hosts.

## 1. Introduction

The perception of bacteria as single-celled primitive life forms is outdated. Single-celled bacteria, known as free-floating planktonic forms, constitute only one small fraction of a bacterial species and primarily serve the purpose of disseminating the bacteria to new habitats [1,2].

There are profound survival advantages to the formation of bacterial communities, such as biofilm [3]. The collaborative effort of sustaining a protected ecosystem allows the bacteria in the biofilm to resist antibiotic treatments [4,5] and to alter their gene expression [6]. As an example, the spirochete *Borrelia*, living in biofilm, does not spend energy on motility [7]. Instead, the bacteria turn down their metabolic activity [8], and as a result, are less sensitive to many types of antibiotics that depend on metabolic processes for bacteriostatic activity [9,10].

Much is known about the immunogenic properties of planktonic bacteria, such as the binding to specific pathogen-associated molecular pattern receptors (PAMPs) on immune cells [11]. The host immune system also mounts a response to the biofilm; however, the response is largely ineffective, as bacteria living in biofilms typically shield themselves by building a matrix that is resilient to attack from immune cells [12]. *Borrelia* biofilm forms an extracellular matrix predominantly composed of alginate [13]. *Pseudomonas aeruginosa* (Pa) and *Staphylococcus simulans* (Ss) produce a slime composed of exopolysaccharides [14]; the slime enhances the bacteria’s ability to infect a host and also creates an envelope that is difficult to penetrate and invade by immune cells. Pa and *Borrelia* biofilms are able to avoid antibiotic treatment by developing into multidrug-tolerant persister cells [15].

*Borrelia* susceptibility to antibiotics was initially studied in acute infections and cultures of free planktonic spirochetes [16], but recent research has extended the focus to include biofilm [17]. The antibiotic susceptibility of *Borrelia burgdorferi* (Bb) to doxycycline showed a significant reduction in the numbers of spirochetal forms, but at the same time triggered a significant and dose-dependent increase in persister cells [9,13], which have reduced metabolic activity and thus have limited detectability microscopically and in diagnostic cultures. 

Natural substances have been investigated for their ability to interfere with multiple aspects of biofilm development, adhesion of the biofilm to surfaces, quorum sensing, and disruption of established biofilm [18,19,20]. Cranberry prevents both bacterial and fungal biofilm formation by decreasing adherence of biofilm during infections of the urinary tract and oral cavity [21,22]. Herbs such as rosemary and peppermint can block the quorum-sensing communication within biofilm [23]. Berberine is a botanical alkaloid with bacteriostatic properties that has also been shown to inhibit biofilm formation and quorum sensing [24,25,26]. N-acetylcysteine, a nutrient that is used to treat mucus in respiratory infections, has been shown to both inhibit new biofilms and destroy existing biofilms [27,28]. From a nutraceutical perspective, there is abundant potential for designing botanical blends with different, complementary mechanisms of action, for a more robust support of biofilm disruption and interference with the bacterial gene expression and metabolic function than any single ingredient alone.

The physical barrier that covers biofilm contributes to immune evasion [12] and antibiotic resistance [17]. Non-pharmaceutical enzymatic treatments have been researched to disrupt biofilm formation by inhibiting new biofilm formation, decreasing bacterial viability, and breaking down the biofilm matrix [29,30]. Pa biofilm can be disrupted by various enzymes such as lipase [31], cellulase [32], and serratiopeptidase [33]. Lysozyme is an antimicrobial enzyme, part of our innate immune system [34]. Other enzymes, including β-glucanase, α-amylase, and protease, were shown to reduce biofilm colonies [35]. It has been suggested that for the disruption of established biofilm to be successful, a multi-enzyme formula including enzymes that can degrade proteins, polysaccharides, microbial DNA, and quorum sensing signals is advantageous [36]. We have previously reported biofilm-disrupting properties in vitro of a nutraceutical enzyme and botanical blend (NEBB), where biofilm treatment resulted in significantly reduced biofilm mass and metabolic activity in Ss and Bb, while the same doses of treatment triggered enhanced slime formation by Pa [37], likely as an evasive tactic to the attack on the biofilm. Our work has continued to document the immunogenic effects of untreated versus disrupted biofilm, with the development of an in vitro methodology reported here. 

**Figure 1:**
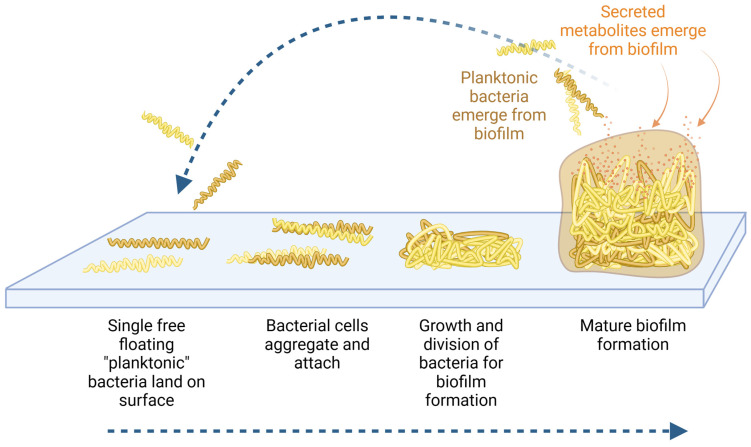
Diagram showing the formation of biofilm where free-floating planktonic bacteria adhere to surfaces and develop a protected community (bottom blue arrow). Within the protected environment of the biofilm matrix, the bacteria undergo epigenetic regulation as they adapt to a sessile lifestyle. From within the protected biofilm, the bacteria produce and secrete many types of metabolites that affect the host biology and immune alertness (orange arrows). Planktonic bacteria may re-emerge from the biofilm (top blue arrow), in search of new habitats.

Bacteria secrete compounds involved in communication, growth regulation through quorum sensing, and toxins to interfere with the host’s immune protection (Figure 1). Bacterial quorum-sensing peptides can affect many biological processes within a mammalian host. Research has focused on characterizing the complete metabolome of the planktonic form of *Borrelia* [38], and various sources of biological material from *Pseudomonas*-infected humans and animals [39]. However, only little is known about the cumulative effects of the secreted metabolites from various bacterial biofilms on the immune function of the mammalian host. We established an experimental model to document such immune effects by harvesting secreted metabolites from established biofilm and adding this crude metabolite fraction to human immune cells in culture. We present results on changes to pro- and anti-inflammatory cytokine production by the human immune cells. In parallel, we tested secreted metabolites from biofilm after treatment with a nutraceutical enzyme and botanical blend (NEBB), previously reported to enhance the protective slime formation by *Pseudomonas* biofilm, while significantly disrupting biofilms from *Staphylococcus simulans* and *Borrelia burgdorferi* in vitro [37]. 

## 2. Materials and Methods

### 2.1. Reagents

Bacterial culture media were purchased from Sigma-Aldrich Inc. (St Louis, MO, USA): Nutrient Broth (Catalogue number 70122) and BSK medium with 6% rabbit serum (Catalogue number B8291). The 96-well culture plates were obtained from Thermo-Fisher Scientific (Waltham, MA, USA): CellStar (Greiner Bio-One, Kremsmünster, Austria, Catalogue number 655-180) for all microbes except *Borrelia burgdorferi* for which collagen-coated 96-well plates were used (Catalogue number 152038). Other reagents were Crystal Violet (Catalogue number V5265, Sigma-Aldrich (St. Louis, MO, USA)) and CyQUANT VyBrant MTT cell viability assay kit (Catalogue number V13154, Invitrogen, Thermo-Fisher Scientific (Waltham, MA, USA)). Roswell Park Memorial Institute 1640 medium, penicillin–streptomycin 100×, phosphate-buffered saline (PBS), and lipopolysaccharide (LPS) were purchased from Sigma-Aldrich Co. (St Louis, MO, USA). Bio-Plex Pro™ human cytokine arrays were purchased from Bio-Rad Laboratories Inc. (Hercules, CA, USA).

### 2.2. Bacterial Strains

Three microbes, known for their ability to live in biofilms, were included in this testing (Table 1). The microbial strains were purchased from the American Type Culture Collection. The recommended culture media for each strain was used, and cultures were performed under conditions that encouraged biofilm formation. The testing for effects of the nutraceutical blend on biofilm disruption involved these steps: (1) culture each microorganism to facilitate biofilm formation in flat-bottom 96-well culture plates; (2) add NEBB and continue culture for 24 h; (3) remove planktonic (free) forms including disrupted biofilm and forms released from biofilm and wash the remaining biofilm with physiological saline; (4) evaluate the estimated mass and metabolic activity of the remaining biofilm in untreated versus treated cultures. 

### 2.3. Nutraceutical Enzyme and Botanical Blend 

The nutraceutical enzyme and botanical blend (NEBB), BioDisrupt™, was provided by the manufacturer, Researched Nutritionals, Los Olivos, CA, USA. The product is a powder that contains water-soluble enzymes, botanical extracts, and N-acetyl cysteine [37]. The powder was used to prepare a suspension in phosphate-buffered saline and allowed to hydrate for 1 h under gentle agitation. Insoluble material was pelleted by centrifugation and the aqueous fraction was filtered through a 0.22-micron filter before adding to cell cultures. Serial dilutions were made in phosphate-buffered saline. 

### 2.4. Removal of Planktonic Forms 

The treatment of established biofilm with NEBB and the resulting disruption of biofilm included the release of planktonic forms into the culture supernatant and the detachment of clumps of bacteria living in biofilm. In order to provide conclusive data on the effects on biofilms, following published methodology [37], planktonic forms had to be removed from the cultures before staining for biofilm mass and metabolic activity. The removal of planktonic forms and the addition of washing buffer were performed using a very low speed to avoid mechanical removal of biofilm material. The removal of planktonic forms was performed using electronic 12-channel pipettes (Viaflo, Integra, Hudson, NH, USA), where the speed was set to “1” (the maximum speed is “10”). Phosphate-buffered saline was added, also using speed “1”, where the liquid was dispensed onto the sidewalls of each well to avoid disruption of biofilm by direct pipetting actions onto biofilm. For the cultures of *Pseudomonas aeruginosa*, this pipetting allowed the scoring of slime formation, where “0” indicated no change in viscosity of the culture medium, and a score of “3” (300%) indicated that the entire culture medium had turned into a mucus plug. 

### 2.5. Harvesting of Biofilm Metabolites

For each treatment condition (untreated versus various doses of NEBB), 9 wells were cultured. After the 24 h culture period, the supernatants were harvested; these supernatants may have contained various pleomorphic forms not living in biofilm, and their metabolites may have contributed to the altered immunogenicity of the metabolites. It was an important part of the methodological rationale to include metabolites from all forms. Therefore, at this point of the bacterial cultures, we did not remove planktonic forms prior to allowing the remaining biofilm to secrete metabolites. This resulted in metabolite fractions that may have contained some compounds from NEBB that were not degraded over the 24 h culture period. From each of the 9 wells, 80 μL supernatant was aspirated, and the supernatants from all 9 wells were pooled. After the supernatants were harvested, the biofilm mass and metabolic activity were evaluated by Crystal Violet and MTT staining, respectively, following a previously published method [37]. Each sample of pooled supernatant was sterile-filtered through a 0.22-micron cellulose acetate syringe filter, and aliquots were prepared into microcentrifuge tubes and frozen at −30 °C until tested for induction of immune-modulating activity in human PBMC cultures. 

### 2.6. Treatment of Human Immune Cells with Bacterial Biofilm Metabolites

**Figure 2:**
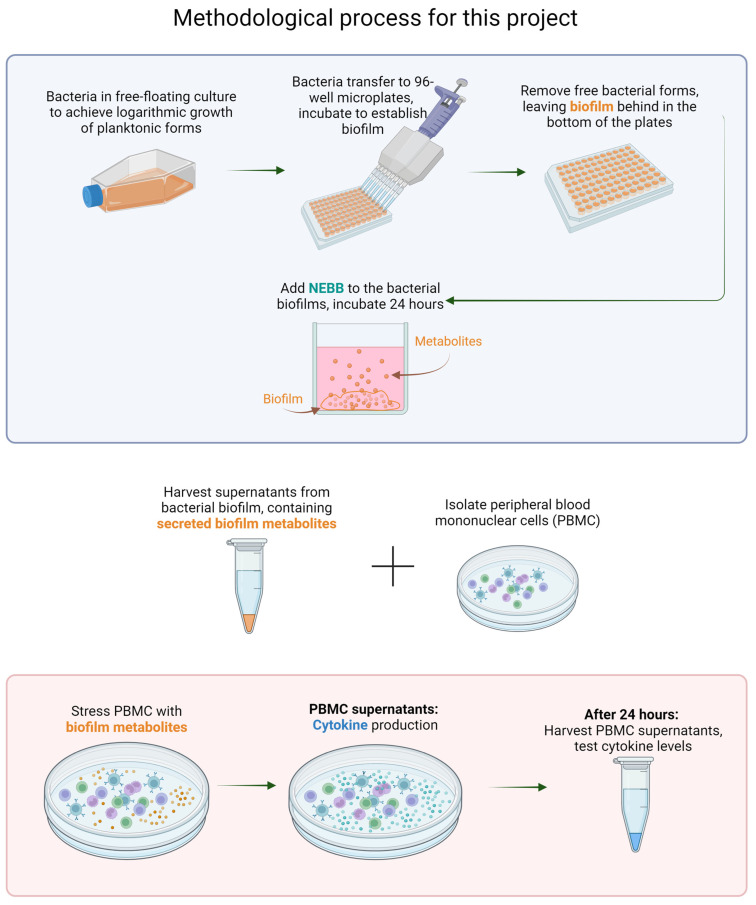
Flow chart showing the methodology used for this research. TOP: Bacterial biofilm is established, and the secreted metabolites from the biofilm cultures are harvested, representing the secretome from mature biofilm after epigenetic changes (differentiating from planktonic forms). BOTTOM: Cultures of human peripheral blood mononuclear cells (PBMC) are exposed to secreted metabolites. The resulting immune activation is measured by testing cytokine levels in the PBMC culture supernatants.

The biofilm supernatants were used for the testing of effects on human immune cells (Figure 2). The thawed biofilm supernatants were vortexed and centrifuged to pellet any solids prior to adding them to human immune cell cultures. Human peripheral blood mononuclear cells were cultured in the absence versus the presence of the bacterial biofilm metabolites. The supernatants from the human white blood cell cultures were tested for the production of 10 pro- and anti-inflammatory cytokines.

### 2.7. Immune Cell Activation

Peripheral blood mononuclear cells (PBMC) were isolated from blood from a healthy human donor upon written informed consent, as approved by the Sky Lakes Medical Center Institutional Review Board, Federalwide Assurance 2603. Blood was drawn into heparin vacutainer vials, and the PBMC were separated from other cell types using Lympholyte Poly (Cedarlane, Burlington, NC, USA), by centrifugation for 35 min at 400× *g*. The PBMC were washed twice in PBS, counted, and the cell suspension adjusted to establish cultures with a cell density at 10^6^/mL, using Roswell Park Memorial Institute 1640 medium containing penicillin–streptomycin. Sterile-filtered biofilm supernatants or LPS (10 ng/mL cell culture) were added to cultures at a volume of 20 μL, and PBMC cultures were then incubated at 37 °C, 5% CO_2_ for 24 h. The highly inflammatory LPS was used as a positive control for immune-cell activation. Untreated negative control cultures consisted of PBMC exposed to phosphate-buffered saline in the absence of bacterial metabolites. All treatments and each positive and negative control were tested in triplicate. After 24 h, supernatants from the PBMC cultures were harvested and kept frozen until subsequent testing for cytokine levels.

### 2.8. Quantification of Cytokine Production by the Immune Cells in Reaction to Biofilm Metabolites

After 24 h of incubation, the supernatants were harvested from the PBMC cultures described above. Levels of 10 cytokines and chemokines were quantified using Bio-Plex protein arrays (Bio-Rad Laboratories Inc.) and utilizing xMAP technology (Luminex, Austin, TX, USA). Interleukin-1 beta (IL-1β), interleukin-1 receptor antagonist (IL-1ra), interleukin-2 (IL-2), interleukin-4 (IL-4), interleukin-6 (IL-6), interleukin-8 (IL-8, CXCL8), interleukin-10 (IL-10), interferon-gamma (IFN-γ), tumor necrosis factor alpha (TNF-α), and macrophage inflammatory protein-1 alpha (MIP-1α, CCL3) were quantified using Bio-Plex protein arrays (Bio-Rad Laboratories Inc.) and the xMAP technology (Luminex, Austin, TX, USA).

### 2.9. Statistical Analysis

Average and standard deviation for each data set was calculated using Microsoft Excel, Version 2404. Statistical analysis of in vitro data was performed using the 2-tailed, independent t-test. Statistical significance was set at *p* < 0.05, and a high level of significance *p* < 0.01.

## 3. Results

### 3.1. Immunomodulatory Activity of Secreted Biofilm Metabolites 

**Figure 3:**
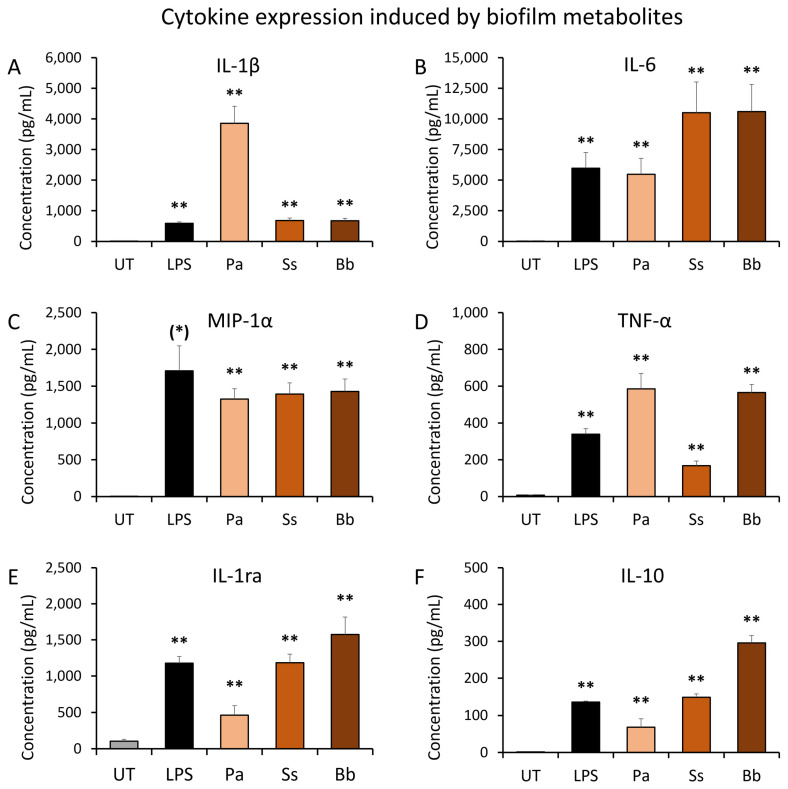
Induction of inflammatory cytokines in cell cultures of human peripheral blood mononuclear cells exposed to bacterial biofilm metabolites. Biofilm metabolites were harvested from established bacterial biofilm cultures of *Pseudomonas aeruginosa* (Pa), *Staphylococcus simulans* (Ss), and *Borrelia burgdorferi* (Bb). Untreated (UT) cultures served as negative controls for cytokine levels in unstimulated PBMC, and lipopolysaccharide (LPS)-treated cultures served as positive controls for inflammatory induction of cytokines. (**A**) Interleukin-1 beta (IL-1β) levels in PBMC cultures. (**B**) Interleukin-6 (IL-6) levels in PBMC cultures. (**C**) Macrophage inflammatory protein-1 alpha (MIP-1α) levels in PBMC cultures. (**D**) Tumor necrosis factor-alpha (TNF-α) levels in PBMC cultures. (**E**) Interleukin-1 receptor antagonist (IL-1ra) levels in PBMC cultures. (**F**) Interleukin-10 (IL-10) levels in PBMC cultures. The levels of statistical significance when comparing the cytokine responses to responses in untreated control cultures are indicated with (*) for *p* < 0.1, * for *p* < 0.05, and ** for *p* < 0.01.

Cultures of peripheral blood mononuclear cells (PBMC) were used to test the effects of secreted metabolites from established bacterial biofilms in vitro (Figure 3). Biofilm metabolites were harvested from established bacterial biofilm cultures of *Pseudomonas aeruginosa* (Pa), *Staphylococcus simulans* (Ss), and *Borrelia burgdorferi* (Bb) and sterile-filtered to ensure that no live bacteria were introduced into the immune cell cultures. Untreated (UT) cultures served as negative controls for cytokine levels in unstimulated PBMC, and lipopolysaccharide (LPS) treated cultures served as positive controls for cytokine induction. 

The results showed that secreted metabolites from bacterial biofilms triggered highly significant changes to immune cell cytokine production in the absence of the bacteria, compared to the untreated control (*p* < 0.01). The cytokine profile showed different patterns for each bacterial species. The biofilm metabolites from Pa biofilm induced 5-fold higher levels of Interleukin-1beta (IL-1β) than secreted metabolites from the other two bacteria as well as LPS (Figure 3A). Pa and Bb metabolites triggered a more robust production of TNF-α than in LPS-treated cultures (Figure 3D). The biofilm metabolites from Pa triggered similar levels of IL-6 as observed in LPS-treated cultures (Figure 3B). Biofilm metabolites from both Ss and Bb triggered approximately 2-fold higher IL-6 levels than seen in Pa metabolite- and LPS-treated cultures (Figure 3B). Additionally, all three bacterial biofilm metabolites triggered MIP-1α production at similar levels (Figure 3C).

In conjunction with these pro-inflammatory cytokines, the anti-inflammatory cytokines interleukin-1 receptor antagonist (IL-1ra) and interleukin-10 (IL-10) were also produced after exposure to biofilm metabolites. The levels were highly significant for all three types of biofilm as well as the LPS control, when compared to untreated control cultures. However, the magnitude of IL-1ra and IL-10 induction varied between the three types of biofilms. When compared to untreated control cultures, the secreted metabolites from Pa triggered lower levels of IL-1ra and IL-10 than LPS, but those from Ss triggered similar levels of IL-1ra and IL-10 as in LPS-treated cultures. In contrast, secreted metabolites from Bb biofilm triggered higher levels of both IL-1ra and Il-10 than metabolites from the other two types of biofilm.

### 3.2. Secreted Metabolites from NEBB-Treated Biofilm Have Immune-Modulating Properties

When biofilm was treated with the nutraceutical enzyme and botanical blend (NEBB), the secreted metabolites from the NEBB-disrupted biofilms triggered altered levels of selected cytokines and chemokines when compared to secreted metabolites from untreated biofilm cultures. The secreted metabolite fractions may have contained some components from NEBB that were not broken down over the 24 h biofilm culture period, and those components from NEBB may have been introduced into PBMC cultures as part of the metabolite fraction. Therefore, the direct effects of NEBB on PBMC cytokine production were documented as a control. 

**Figure 4:**
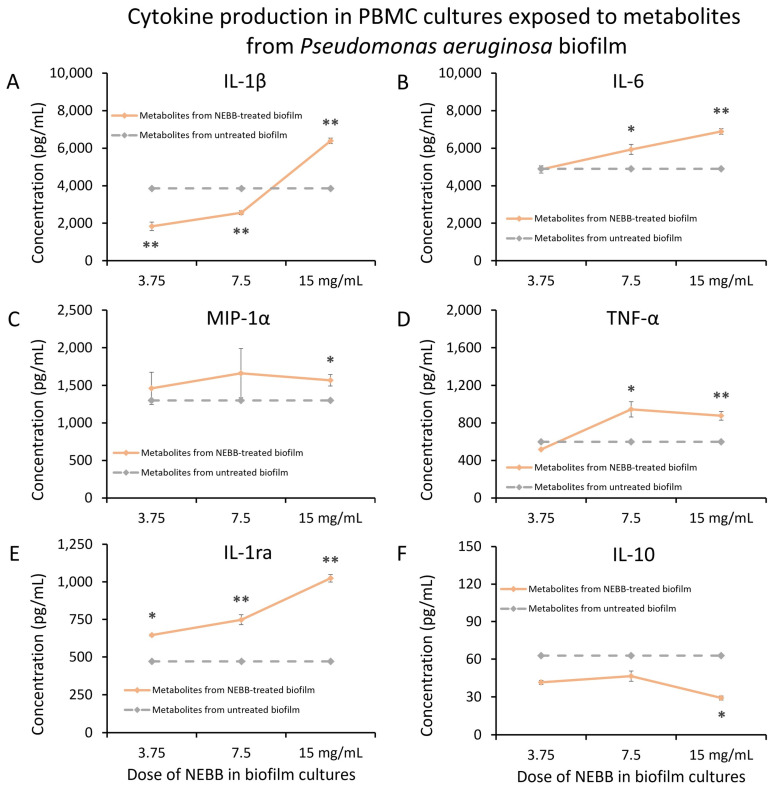
Cytokine levels in cultures of human peripheral blood mononuclear cells exposed to secreted metabolites from NEBB-treated *Pseudomonas aeruginosa* (Pa) biofilm. Solid lines show the cytokine levels triggered by metabolites from NEBB-treated Pa biofilm cultures, where the doses refer to the amount of NEBB in the Pa bacterial biofilm cultures before harvesting the secreted metabolites. The cytokine levels induced by metabolites from untreated Pa biofilm are shown as dashed grey lines, based on data from Figure 3. For reference, the direct effects of NEBB on PBMC cytokine production are shown by dotted green lines, where the doses refer to the amount of NEBB in PBMC cultures. The levels of statistical significance when comparing cytokine responses in PBMC cultures treated with metabolites from NEBB-treated Pa biofilm to responses in PBMC cultures treated with metabolites from untreated Pa biofilm are shown as * for *p* < 0.05 and ** for *p* < 0.01. (**A**) Interleukin-1 beta (IL-1β) levels in PBMC cultures showed a bi-phasic response to metabolites from NEBB-treated Pa biofilm, with a highly significant increase in biofilm treated with a high dose of NEBB, while lower doses of NEBB triggered reduced levels of IL-1β compared to metabolites from untreated Pa biofilm. (**B**) Interleukin-6 (IL-6) levels in PBMC cultures showed a dose-related increase in IL-6 levels, proportional to the dose of NEBB used to treat the Pa biofilm, reaching a high level of significance for the highest dose of NEBB (*p* < 0.01). (**C**) Macrophage inflammatory protein-alpha (MIP-1α) levels in PBMC cultures were mildly increased by metabolites from NEBB-treated Pa biofilm, compared to metabolites from untreated Pa biofilm; however, it was inconclusive whether this may have been a result of residual NEBB in the metabolite fraction when harvested since NEBB alone triggered a similar mild increase in MIP-1α. (**D**) Tumor necrosis factor-alpha (TNF-α) levels in PBMC cultures were mildly increased by metabolites from NEBB-treated Pa biofilm, compared to metabolites from untreated Pa biofilm, reaching a high level of significance for the highest dose of NEBB (*p* < 0.01). (**E**) Interleukin-1 receptor antagonist (IL-1ra) levels in PBMC cultures showed a dose-related increase by metabolites from NEBB-treated Pa biofilm, compared to metabolites from untreated Pa biofilm, reaching a high level of significance for the two highest doses of NEBB (*p* < 0.01). However, it remains inconclusive whether the effect at the two lower doses may have been a result of residual NEBB in the metabolite fraction when harvested since NEBB alone triggered a similar mild increase in IL-1ra at those doses. Therefore, only the effect at the highest dose is conclusive. (**F**) Interleukin-10 (IL-10) levels in PBMC cultures were reduced by metabolites by NEBB-treated Pa biofilm, compared to metabolites from untreated Pa biofilm. Due to the direct effects of NEBB on PBMC production of IL-10, this effect cannot be conclusively attributed to the secreted bacterial metabolites.

*Pseudomonas aeruginosa* (Pa) biofilm was treated with NEBB for 24 h across the dose range of 3.75–15 mg NEBB/mL bacterial culture where a robust and highly significant increase in biofilm metabolic activity was previously documented [37]. The NEBB treatment triggered changes in the effects of the bacterial metabolites on human immune cells in culture. At the highest dose of NEBB, where the Pa biofilm showed the highest increase in metabolic activity, the secreted metabolites showed the strongest immune activating properties, including highly significant increases in the immune cell production of IL-1β (Figure 4A), IL-6 (Figure 4B), and TNF-α (Figure 4D) in PBMC cultures when compared to the cytokine levels induced by metabolites from untreated Pa biofilm.

In addition, secreted metabolites from NEBB-treated Pa biofilm triggered significant changes to the anti-inflammatory cytokines IL-1ra (Figure 4E). The secreted metabolites from NEBB-treated Pa biofilm induced increased levels of IL-1ra, which at the highest dose of NEBB was more than that of secreted metabolites from untreated Pa biofilm (Figure 4E). In contrast, the secreted metabolites from NEBB-treated Pa biofilm triggered reduced levels of IL-10 production in PBMC cultures when compared to the cytokine levels induced by metabolites from untreated Pa biofilm (Figure 4F).

**Figure 5:**
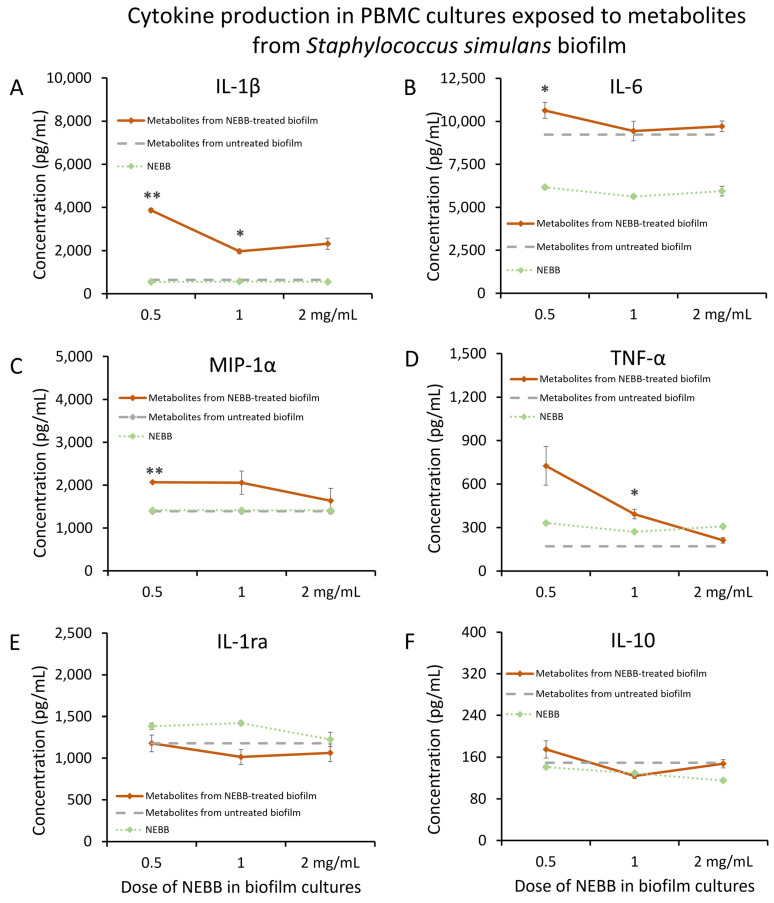
Cytokine levels in cultures of human peripheral blood mononuclear cells exposed to secreted metabolites from NEBB-treated *Staphylococcus simulans* (Ss) biofilm. Solid lines show the cytokine levels triggered by Ss metabolites from NEBB-treated biofilm cultures, where the doses refer to the amount of NEBB in the bacterial biofilm cultures before harvesting the secreted metabolites. The cytokine levels induced by metabolites from untreated Ss biofilm are shown as dashed grey lines, based on data from Figure 3. For reference, the direct effects of NEBB on PBMC cytokine production are shown by dotted green lines, where the doses refer to the amount of NEBB in PBMC cultures. The levels of statistical significance when comparing cytokine responses in PBMC cultures treated with metabolites from NEBB-treated Ss biofilm to responses in PBMC cultures treated with metabolites from untreated Ss biofilm are shown as * for *p* < 0.05 and ** for *p* < 0.01. (**A**) Interleukin-1 beta (IL-1β) levels in PBMC cultures treated with metabolites from NEBB-treated Ss biofilm showed robust increases, compared to metabolites from untreated Ss biofilm, reaching significance for the middle dose of NEBB (*p* < 0.05) and a high level of significance for the lowest dose of NEBB (*p* < 0.01). (**B**) Interleukin-6 (IL-6) levels in PBMC cultures treated with metabolites from NEBB-treated Ss biofilm showed a minor increase, compared to metabolites from untreated Ss biofilm, reaching significance for the lowest dose of NEBB (*p* < 0.05). (**C**) Macrophage inflammatory protein-alpha (MIP-1α) levels in PBMC cultures treated with metabolites from NEBB-treated Ss biofilm were increased, compared to metabolites from untreated Ss biofilm, reaching a high level of significance for the lowest dose of NEBB (*p* < 0.01). (**D**) Tumor necrosis factor-alpha (TNF-α) levels in PBMC cultures treated with metabolites from NEBB-treated Ss biofilm were increased, compared to metabolites from untreated Ss biofilm, reaching significance for the middle dose of NEBB (*p* < 0.05). (**E**) Interleukin-1 receptors antagonist (IL-1ra) levels in PBMC cultures treated with secreted metabolites from NEBB-treated Ss biofilm were not significantly different from PBMC cultures treated with metabolites from untreated Ss biofilm. (**F**) Interleukin-10 (IL-10) levels in PBMC cultures were not majorly affected by metabolites from NEBB-treated Ss biofilm, compared to metabolites from untreated Ss biofilm.

*Staphylococcus simulans* (Ss) biofilm was treated with NEBB for 24 h at the dose range of 0.5–2.0 mg NEBB/mL bacterial culture, where the biofilm showed disruption and increased metabolic activity [37]. Metabolites secreted from NEBB-treated Ss biofilm in these cultures triggered significant increases in the immune cell production of IL-1β (Figure 5A), IL-6 (Figure 5B), and TNF-α (Figure 5D), as well as the chemokine MIP-1α (Figure 5C), particularly at lower doses, when compared to the cytokine levels induced by untreated Ss biofilm.

In contrast, secreted metabolites from NEBB-treated Ss biofilm did not trigger major changes to the anti-inflammatory cytokines IL-1ra (Figure 5E) or IL-10 (Figure 5F). 

**Figure 6:**
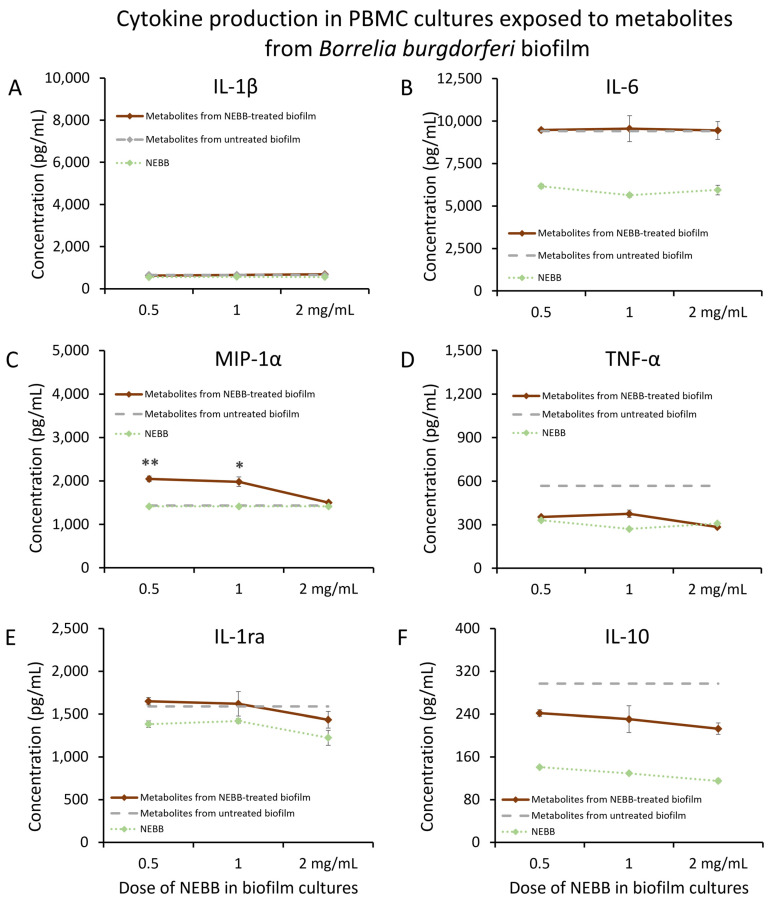
Cytokine levels in cultures of human peripheral blood mononuclear cells exposed to secreted metabolites from NEBB-treated *Borrelia burgdorferi* (Bb) biofilm. Solid lines show the cytokine levels triggered by Bb metabolites from NEBB-treated biofilm cultures, where the doses refer to the amount of NEBB in the bacterial biofilm cultures before harvesting the secreted metabolites. The cytokine levels induced by metabolites from untreated Bb biofilm are shown as dashed grey lines, based on data from Figure 3. For reference, the direct effects of NEBB on PBMC cytokine production are shown by dotted green lines, where the doses refer to the amount of NEBB in PBMC cultures. The levels of statistical significance when comparing cytokine responses in PBMC cultures treated with metabolites from NEBB-treated Bb biofilm to responses in PBMC cultures treated with metabolites from untreated Bb biofilm are shown as * for *p* < 0.05 and ** for *p* < 0.01. (**A**) Interleukin-1 beta (IL-1β) levels in PBMC cultures treated with secreted metabolites from NEBB-treated Bb biofilm were similar to levels in PBMC cultures treated with metabolites from untreated Bb biofilm. (**B**) Interleukin-6 (IL-6) levels in PBMC cultures treated with secreted metabolites from NEBB-treated Bb biofilm were similar to the levels in PBMC cultures treated with metabolites from untreated Bb biofilm. (**C**) Macrophage inflammatory protein-alpha (MIP-1α) levels in PBMC cultures treated with metabolites from NEBB-treated Bb biofilm were increased, compared to metabolites from untreated Bb biofilm, reaching a high level of significance for the lowest dose of NEBB (*p* < 0.01). (**D**) Tumor necrosis factor-alpha (TNF-α) levels in PBMC cultures treated with metabolites from NEBB-treated Bb biofilm were robustly reduced, compared to metabolites from untreated Bb biofilm. However, it was inconclusive whether this may have been a result of residual NEBB in the metabolite fraction when harvested since NEBB alone triggered a similar mild increase in TNF-α and MIP-1α. (**E**) Interleukin-1 receptors antagonist (IL-1ra) levels in PBMC cultures treated with secreted metabolites from NEBB-treated Bb biofilm were similar to the levels in PBMC cultures treated with metabolites from untreated Bb biofilm. (**F**) Interleukin-10 (IL-10) levels in PBMC cultures treated with metabolites from NEBB-treated Bb biofilm were reduced, compared to metabolites from untreated Bb biofilm. However, it was inconclusive whether this may have been a result of residual NEBB in the metabolite fraction when harvested, since NEBB alone triggered a decrease in IL-10, and this reduction cannot be conclusively attributed to the secreted bacterial metabolites.

Mature biofilm from *Borrelia burgdorferi* (Bb) was treated with NEBB for 24 h at the dose range of 0.5–2.0 mg NEBB/mL bacterial culture, where the biofilm showed disruption and increased metabolic activity. Metabolites secreted from NEBB-treated Bb biofilm triggered highly significant increases in the immune cell production of the chemokine MIP-1α compared to metabolites from untreated biofilm (Figure 6C), particularly at lower doses, but did not trigger any changes to IL-1β (Figure 6A) or IL-6 (Figure 6B). Mild reductions in TNF-α levels were seen in PBMCs treated with metabolites from NEBB-treated Bb biofilm cultures compared to that of metabolites from untreated Bb biofilm (Figure 6D). In addition, no difference was seen in the levels of IL-1ra in PBMC cultures treated with and without NEBB-treated Bb biofilm metabolites (Figure 6E). However, the secreted metabolites from NEBB-treated Bb biofilm triggered a reduction in IL-10 production in PBMC cultures, compared to metabolites from untreated Bb biofilm (Figure 6F); due to the direct effects of NEBB on PBMC production of IL-10, this effect cannot be conclusively attributed to the secreted bacterial metabolites.

## 4. Discussion

**Figure 7:**
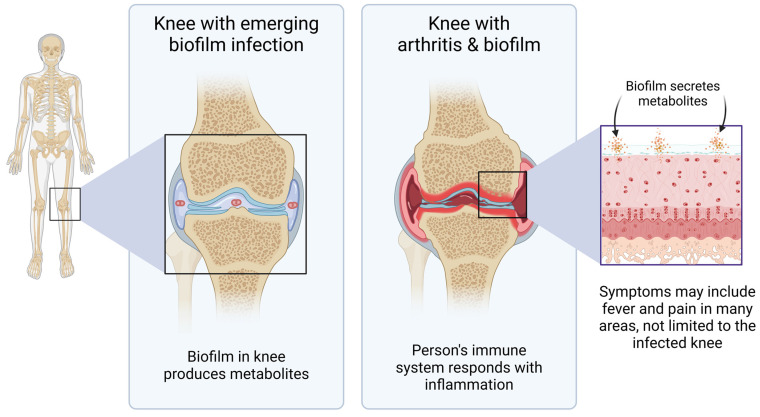
Diagram showing an example of clinical importance of understanding the biology of pathogenic organisms living in biofilm. This example depicts a joint where bacterial biofilm establishes communities from which they can secrete metabolites that affect the host, not only in the affected anatomical areas, but systemically.

Pathogenic bacterial biofilm uses many simultaneous communication methods to create its habitat, communicate within and between biofilms, protect itself from attack from the host immune system, and dysregulate the host immune defense. Biofilm can divert the immune alertness in part by secreting products that actively suppress recruitment of immune cells to the biofilm-affected tissues [40], while at the same time continuing to sustain chronic inflammation in the host [41]. Bacteria living in biofilm communicate within the biofilm by quorum sensing. In addition, extracellular polymeric substances form the biofilm matrix and contribute to the unique characteristics of the virulence of the biofilm. Biofilm communicates with the host through secreted metabolites (Figure 7), where in this context the metabolites are defined as any substance produced by the bacteria and escaping the encapsulated biofilm matrix, thereby entering the host tissue fluids, lymphatics, blood circulation, and in many situations, also crossing the blood–brain barrier [42], with systemic consequences for the infected host, where the disease symptoms are complex, driven not only by the epigenetic state of a given bacterial species but also by the habitat and host responses in the affected tissues and organs. 

Whereas specific secreted compounds have been studied from acute infections [43,44] and from established bacterial biofilm [45,46], little is known about the effects on the host immune system when exposed to the totality of secreted metabolites from established bacterial biofilm. The method we present here is intentionally designed to reflect the typical situation of time and spatial separation between established biofilm in tissue such as joints, lungs, or skin, and the host immune cells responding to its presence. This model is different from co-cultures of free planktonic bacteria with host immune cells, which resemble the acute phase of infection before the bacteria form biofilm. Our experimental model involved the complexity of peripheral blood mononuclear cells, reflecting the naturally occurring mix of monocytes/macrophages, T and B lymphocytes, dendritic cell types, natural killer cells, and stem cells. We suggest that this model is physiologically and clinically relevant, as it is representative of the cellular community in tissues such as the gut, skin, muscles, and lungs. This collaborative effort between multiple immune cells is further illustrated by the ability of PBMC cultures to produce IL-10 in response to the LPS inflammatory control [47,48], in contrast to the THP-1 cell line that is not able to do so [48].

Our results from using this model on established biofilm from three different bacterial species have shown that the secreted metabolites generated from all three types of biofilms were highly inflammatory and triggered robust production of pro-inflammatory cytokines by human immune cells. The cytokine profiles showed differences between the bacterial species, where Pa triggered manifold higher levels of IL-1β than the other two bacterial species. The predominance of IL-1β mirrors what has been reported clinically using respiratory isolates of Pa isolated during the early stages of Pa infections from patients with cystic fibrosis, whereas the increase in IL-6 and TNF-α has parallels to what was seen in chronic Pa infections [49]. The clinical implication of these cytokine shifts as the infection becomes chronic was suggested to reflect an adaptive change, resulting in reduced macrophage cell death and increased inflammatory cytokine production, affecting lung function and disease progression [49].

The changed immune response to secreted metabolites from disrupted Ss and Bb biofilm included a significant increase in the metabolites’ ability to increase the production of the chemokine MIP-1α from immune cells, suggesting increased recruitment of phagocytic cells to the biofilm-affected tissue. In cases where the biofilm is disrupted, this may allow an improved immune recognition of the bacteria. The downstream effects of the increased recruitment of immune cells to the biofilm area may be a beneficial reduction in biofilm load in the host, but the enhanced bacterial killing is likely associated with fever and malaise. Increased immunological attack on spirochetal bacteria released from disrupted biofilm can cause the Jarisch–Herxheimer reaction [50], which is often transient and a sign of active immune defense activity. Our data on the disruption of NEBB-treated biofilm [37] and the altered immunogenicity of NEBB-treated biofilm reported here showed a species-selective change to pro- and anti-inflammatory immune response. The immune-evasive tactics of Bb biofilm are known to include induction of IL-10 as a tactic to reduce the chemotactic and phagocytic activity of macrophages. We showed that immune cells exposed to the secreted metabolites from Bb biofilm produced 2-fold higher levels of the anti-inflammatory cytokine IL-10 than immune cells exposed to secreted metabolites from Ss metabolites, and 4-fold higher IL-10 levels than immune cells exposed to Pa metabolites. It was previously shown that co-cultures of purified mouse macrophages and dendritic cells with planktonic forms of Bb triggered increased production of IL-10 by the immune cells, which in turn suppressed phagocyte-recruiting chemokine signals and phagocytosis [51]. Therefore, our observation that secreted metabolites from Bb biofilm also induced IL-10 production by immune cells suggests that this effect is likely mediated by secreted messengers and does not require the presence of the free spirochete. A similar effect was seen for the anti-inflammatory cytokine IL-1ra, where secreted metabolites from the Bb biofilm triggered much higher levels than metabolites from the Pa or Ss biofilm. 

The experimental model presented here is relevant for the documentation of communication between established biofilm and the host immune system, both during chronic persistent biofilm infections, and during treatments to disrupt the established biofilm. This model is less applicable for the initial infection by planktonic forms with a different epigenetic programming [52]. Also, in the case of Bb infection, experimental models for acute infections need to consider the components in the saliva of the infection-transmitting arthropod [53]. Our methodology may be adapted to co-cultures of established biofilm with immune cells, thereby mimicking aspects of the in vivo situation when host immune cells receive chemotactic signals and migrate to the biofilm tissue. The Pa and Bb biofilm-forming bacteria presented in this paper are also known to be capable of establishing intracellular infections [54,55]. This may possibly be in the form of biofilm-like colonies inside the cytoplasm or organelles of host cells [56], or even inside the lipid bilayer cell membrane of the host cells [57].

The results reported here are preliminary, and further work is needed. Further research directions include many similar aspects of biofilm research into the communication between established biofilm and the human host, including biofilms associated with gut mucosa, as well as bacterial biofilms in tissue such as joint cartilage [55], lungs [54], and skin [58,59]. Our experimental model included the complete secretome from each biofilm culture, likely in combination with components released from the biofilm matrix, such as non-encoded non-protein metabolites and extracellular DNA. We monitored the immune responses using a physiologically relevant complex community of immune cell types, serving as a starting point for screening the potential cumulative effects of the totality of secreted compounds from pathogenic biofilm. Much work remains to be completed, including metabolomic profiling. Furthermore, multi-species biofilm seems to have developed symbiotic relationships, and the study of the secretome from multi-species biofilm, such as Bb cohabiting with *Helicobacter pylori* in Morgellon’s disease [58], and with *Chlamydia* in multiple cases of *Borrelia* lymphocytoma [59], is in urgent need of further research.

## 5. Conclusions

There is a pressing need for effective combinatorial treatment of biofilm in chronic illnesses such as cystic fibrosis and Lyme borreliosis. Recent research has focused on the uses of inhibitors and modulators of quorum-sensing signal molecules in the management of chronic illnesses associated with biofilm. Our preliminary result reported here has helped document that enzymatic disruption of the biofilm matrix, combined with botanical interference with bacterial epigenetics triggered species-specific changes to secreted metabolites, and thereby caused altered immune reactions. Key findings include documentation of increased production of the chemokine MIP-1α, known to assist in the recruitment of phagocytes to the area, combined with reducing the immune-evasive tactics of biofilm after nutraceutical disruption via reduced IL-10 production, most prominent for the disrupted Bb biofilm. Further clinical research into the effects on pro- and anti-inflammatory cytokine levels in patients with chronic illnesses related to bacterial biofilm in the context of the consumption of specific nutraceutical blends is needed.

## Figures and Tables

**Table 1:** Microbial strains.

Microbial Strain	Gram Staining	LPS ^1^
*Pseudomonas aeruginosa* (Schroeter) Migula (ATCC^®^ 9027)	Negative	Yes
*Staphylococcus simulans* Kloos and Schleif-er (ATCC^®^ 11631) ^2^	Positive	
*Borrelia burgdorferi* Strain B31 (ATCC^®^ 35210)	Negative	No

^1^ LPS: Lipopolysaccharide. ^2^ Coagulase-negative, penicillin-resistant.

## Data Availability

The raw data supporting the conclusions of this article will be made available by the authors on request.
